# Recent Advances in Antibiofouling Materials for Seawater-Uranium Extraction: A Review

**DOI:** 10.3390/ma16196451

**Published:** 2023-09-28

**Authors:** Peng Liu, Minyan An, Teng He, Ping Li, Fuqiu Ma

**Affiliations:** 1 Yantai Research Institute and Graduate School, Harbin Engineering University, Yantai 264000, China; liup@hrbeu.edu.cn (P.L.); anminyan@hrbeu.edu.cn (M.A.); heteng@hrbeu.edu.cn (T.H.); 2 College of Nuclear Science and Technology, Harbin Engineering University, Harbin 150001, China; 3 Northwest Institute of Eco-Environment and Resources, Chinese Academy of Sciences, Lanzhou 730000, China; liping@lzb.ac.cn

**Keywords:** antibiofouling, seawater-uranium extraction, adsorption, antibacterial

## Abstract

Nuclear power has experienced rapid development as a green energy source due to the increasing global demand for energy. Uranium, as the primary fuel for nuclear reactions, plays a crucial role in nuclear energy production, and seawater-uranium extraction has gained significant attention. However, the extraction of uranium is usually susceptible to contamination by microorganisms, such as bacteria, which can negatively affect the adsorption performance of uranium adsorption materials. Therefore, an important challenge lies in the development of new antibacterial and antiadhesion materials to inhibit the attachment of marine microorganisms. These advancements aim to reduce the impact on the adsorption capability of the adsorbent materials. This paper reviews the antibiofouling materials used for extracting seawater uranium, and corresponding mechanisms are discussed.

## 1. Introduction

With our country’s goal of “carbon neutrality and carbon peaking”, nuclear energy, as a relatively clean energy, has developed rapidly due to its low carbon emissions, causing the demand for nuclear energy continue to grow [1]. Uranium is the main raw material for nuclear reactions, but the reserves of uranium resources on land are limited, and the exploitable uranium resources can only last for several decades [2]. The uranium content in seawater is as high as 4.5 billion tons, which are hundreds of times that on the land reserves. Therefore, the efficient recovery of uranium in seawater is crucial for the sustainable development of the nuclear industry [3,4]. The extraction methods for uranium include the adsorption method [5], chemical precipitation method [6], membrane-separation method [7], ion-exchange method [8], photocatalysis [9], etc. Due to the complex environment of seawater, it is still a huge challenge to extract uranium from seawater in order to achieve engineering applications. The ultralow uranium concentration (3.3 ppb) in seawater and the serious biofouling caused by marine organisms limit the uranium extraction [10,11,12]. Pollution by marine organisms reduces the adsorption performance of uranium adsorption materials by 30% [13], and will surely increase the economic costs of seawater-uranium extraction. Wang et al. [14] prepared a bifunctional polymer polypeptide hydrogel (PPH-OP). Its main component is a biosafe omiganan polypeptide (OP), which not only has high-antibacterial activity, but also can closely bind uranium species in seawater. The cost of PPH-OP for uranium extraction is about USD 170.07/kg-U, which is much lower than the currently reported uranium adsorbents (USD 204.91–293.22/kg-U). Therefore, the use of PPH-OP to extract uranium from seawater has certain economic feasibility. Ma et al. [15] prepared polyamidoxime (AO-OpNpNc) fiber adsorption materials with an interconnected open-pore structure, nanoparticles, and nanochannels by two-step grafting polymerization. The cost of uranium extraction from seawater using AO-OpNpNc fiber can be reduced to USD 80.70–86.25/kg-U, which is comparable to the international uranium price. This new type of adsorption material for uranium extraction from seawater is expected to realize the industrialization of uranium extraction from seawater. Therefore, it is of great strategic significance to study materials with a good antibiofouling ability and excellent adsorption performance for U(VI) in seawater.

**Figure 1:**
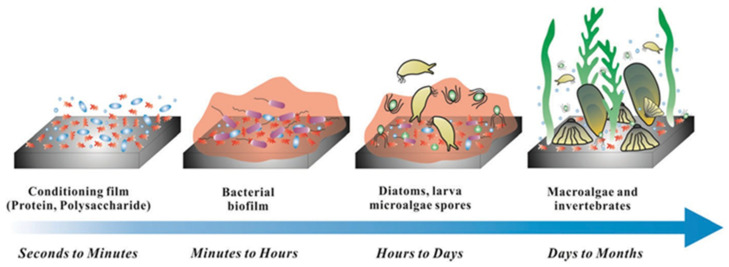
Schematic diagram of material biocontamination [23].

Marine biofouling is the accumulation of microorganisms, algae, plants, or animals on the surface of a material. In the marine environment, biofouling is generally divided into five steps [16,17,18,19,20]. In the first stage, after the material is immersed in seawater for a few seconds, organic molecules will adhere to the surface of the material to form a conditioning film; in the second stage, microbial cells attach to the surface of the material and fix the bacteria on the surface; in the third stage, the surface of the material secretes adsorbed proteins and begins to multiply and secrete the peptidoglycan layer to form a microbial film; in the fourth stage, the rough biofilm surface begins to capture more particles and other small organisms; in the final stage, marine macrobiotics, such as mussels, barnacles, or algae, grow on the surface of the contaminated material (Figure 1). Due to the complex marine environment, there are many kinds of organisms in the ocean, which have a great influence on the adsorption performance of uranium extraction from seawater. Therefore, while pursuing adsorption efficiency, more attention should be paid to the comprehensive properties of materials, and adsorption materials with broad-spectrum antibacterial properties should be paid attention to. Nanometal-based materials have attracted the attention of researchers due to their excellent antibacterial properties and low induced bacterial resistance [21]. Recently, Lu et al. [22] prepared a novel antibacterial copper-cluster molecule (CuCs) based on theanine peptide. The CuCs exhibit a strong broad-spectrum antibacterial ability. Its antibacterial effect is not only to destroy the cell wall and cell membrane structure of bacteria, but also to regulate the ratio of glutathione/oxidized glutathione (GSH/GSSG) by inhibiting the activity of glutathione reductase, thereby causing the outbreak of ROS and eventually leading to bacterial death.

The ideal antibiofouling material can provide a surface free of biological attachment for a certain period of time, which can prevent marine organisms from affecting the adsorption effect. The basic principles of antifouling can be divided into two types: one is antibacterial antibiological pollution materials, which can release substances that can kill or interfere with the growth of marine larvae, preventing bacteria and organisms from growing on its surface; the other is an antiadhesion antibiofouling material. According to the selectivity of fouling organisms to the surface structure of adsorbed materials, materials with certain surface physical properties are developed. For example, superhydrophilic materials can prevent marine organisms from attaching to their surfaces [24,25].

## 2. Antibacterial Materials

According to the source and chemical composition of the material, the current common antibacterial uranium adsorption materials can be divided into the following materials.

### 2.1. Inorganic Metal Particle Antibacterial Material

Inorganic antibacterial materials were developed in the 1960s, with broad-spectrum and efficient antibacterial activity and durability. At the same time, inorganic materials as adsorption materials have the advantages of simple preparation, a large specific surface area, and easy adsorption. Therefore, inorganic metal materials are widely used in the field of uranium extraction from seawater [26]. The antibacterial mechanism of inorganic metal materials is mainly divided into two types. One is that the ions on the metal materials will be released into the solution. These metal ions can adsorb on the cells, destroy the peptidoglycan cell wall of the bacteria, and also interact with the proteins in the bacterial cells so that the cells lose the ability to proliferate and die, so as to achieve the purpose of long-term sterilization. The other is that metal ions produce reactive oxygen free radicals to kill bacteria, and the cell wall of bacteria will be destroyed by reactive oxygen species to inhibit the reproduction of bacteria [27,28,29,30].

**Figure 2:**
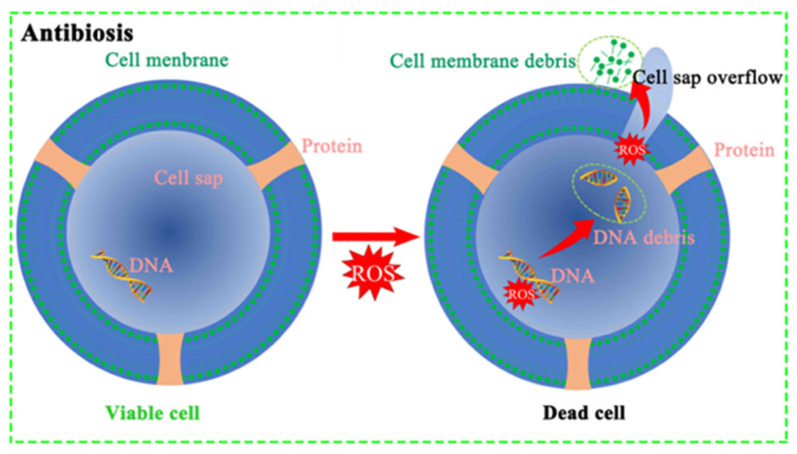
Schematic diagram of antibacterial activity of FF-Cu_2_O/AO [31].

Pu et al. [31] synthesized Cu_2_O particles on the surface of waste-feather fibers by in situ synthesis, and compounded them with amidoxime groups to prepare FF-Cu_2_O/AO, which was used to improve the antifouling activity and uranium-adsorption efficiency (Figure 2). The inhibition zones of FF-Cu_2_O/AO to *Escherichia coli* (Gram-negative bacteria), *Vibrio vulnificus*, and *Vibrio alginolyticus* (marine bacteria) were 26.42 ± 0.43 mm, 38.78 ± 0.65 mm, and 33.47 ± 0.55 mm, respectively. The maximum adsorption capacity of FF-Cu_2_O/AO can reach 816 mg/g (pH = 7.0, 8.0 ppm). After soaking in natural seawater for 30 days, the uranium-adsorption capacity of FF-Cu_2_O/AO was 7.73 mg/g. Sun et al. [32] loaded Co single atoms on the polyacrylamide oxime (PAO) material PAO-Co, which could catalyze the generation of reactive oxygen species (ROS) and inhibit the growth of various microorganisms, and the inhibition rate ranged from 77.0% to 93.4%. The highest uranium adsorption capacity of PAO-Co in uranium-containing simulated seawater was 366 mg/g (pH = 5.0, 8.0 ppm). After four regenerations, the material retained more than 70% of the initial uranium-adsorption capacity and 90% of the initial antibacterial activity, respectively. After soaking in natural seawater containing biofouling for 49 days, it had a uranium-adsorption capacity of 9.7 mg/g, which is only 11% lower than that of natural seawater without biofouling.

### 2.2. Organic Antibacterial Materials

The organic antibacterial material is typically obtained by grafting the antibacterial functional group to the adsorbents. The obtained adsorbents used for seawater-uranium extraction usually have the characteristics of high mechanical strength and good stability. To date, various organic antibacterial materials have been developed, including guanidines, quaternary ammonium salts, quaternary phosphonium salts, pyridines, phenols, imidazoles, and organic halides [33,34,35,36]. Organic antibacterial materials mainly destroy the integrity of bacterial cell membranes by binding to anions on the cell surface, thereby inhibiting bacterial activity. Among them, quaternary ammonium salt is the most widely used antibacterial agent. The quaternary ammonium salt is positively charged by its nitrogen atom and can bind to negatively charged cells, thereby damaging the cell wall and cell membrane of bacteria and causing bacterial death [37]. He et al. [38] introduced polyhexamethylene biguanidine (PHMB)-modified acrylic fiber to obtain the PAO-PHMB-A adsorption material. In seawater with a bacterial content of 10^9^ CFU/mL, the antibacterial activity was as high as 99.71%. At a pH = 8.0, the adsorption capacity of the PAO-PHMB-A material was 525.89 mg/g. At the same time, it had good salt tolerance. At the NaCl concentration of 0.44 M, the adsorption capacity of PAO-PHMB-A was 379.33 mg/g. For 30 days in natural seawater, the uranium adsorption capacity of PAO-PHMB-A was 3.19 mg/g. Yi et al. [39] used guanidine-modified zeolite molecular sieve 13X (ZMS-G) as a carrier to prepare the uranium (VI)-ion-imprinting adsorbent IIZMS-G containing phosphine ligands. The inhibitory rates of IIZMS-G to *Escherichia coli* and *Staphylococcus aureus* were 99.99% and 98.96%, respectively. The maximum adsorption capacity of IIZMS-APTES for uranium was 234.74 mg/g. After five cycles, the adsorption capacity of IIZMS-G changed from 138.54 mg/g to 110.84 mg/g, while the removal rate remained above 80%.

Phosphorylation reagents are often used as extraction reagents for U(VI) separation. The synthesized phosphorus-containing ligands not only have a specific affinity for uranyl ions, but also have excellent broad-spectrum antibacterial properties [40]. It is a better way to improve the antifouling performance of PAO-based materials by introducing phosphate groups into the surface of PAO-based materials through surface coating, surface grafting, plasma treatment, and chemical treatment [41]. Vinylphosphonic acid (VPA, CH_2_CH-PO_3_H_2_) is an organic synthesis intermediate with a simple structure and low toxicity. Its polymer polyvinyl phosphate (PVPA) has been widely used in the field of medicine. In order to improve the antibiofouling performance and adsorption capacity of PAO, He et al. [42] modified PVPA on the surface of PAO. VPA was polymerized on the surface of polyacrylonitrile (PAN) by N_2_ plasma technology. Then, hydroxylamine hydrochloride was added for the amidoxime treatment, and the cyano group was converted to the amidoxime group to obtain the PVPA/PAO adsorbent. The adsorption equilibrium was reached within 24 h. At a pH = 8.2 and 298 K, the maximum adsorption capacity was 145 mg/g. With *Vibrio alginolyticus* from 0 to 4.45 × 10^4^ CFU/mL, the recovery rate of PVPA/PAO to U(VI) was 23.0% (from 27.0% to 20.8%), which has excellent antibiological contamination performance.

Organic antibacterial seawater-uranium-extraction material has become the main research direction of seawater-uranium-extraction material at present because of its fast sterilization speed and strong sterilization ability. Organic antibacterial materials still have many limitations in the extraction of uranium from seawater. The synthesis conditions of organic antibacterial materials are harsh and unstable in seawater. The extensive use of organic antibacterial agents will lead to bacterial resistance and reduce the antibacterial effect. It also has poor heat resistance, it is easy to decompose, and causes pollution. Moreover, the safety is poor, and the organic antibacterial agent has cytotoxicity, which may potentially harm ecological environments.

### 2.3. Natural Antibacterial Materials

**Figure 3:**
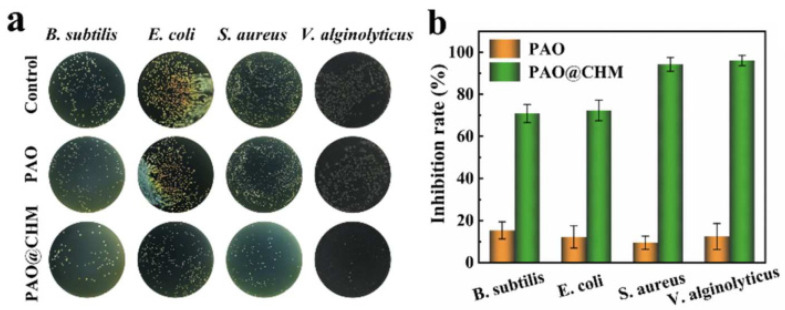
(**a**) Antibacterial spectrum and (**b**) antibacterial activity of PAO and PAO@CHM [45].

Natural antibacterial materials come from the extracts of natural substances. The substances with antibacterial properties in organisms or other natural substances are extracted by separation, purification, and other steps. Natural antibacterial materials have the advantages of good biocompatibility, light pollution to the environment, and low toxicity. However, natural antibacterial agents have a short antibacterial time and are susceptible to many factors, such as the concentration and pH [43]. According to different sources, natural antibacterial materials are divided into three types: plant-derived, animal-derived, and microbial-derived natural antibacterial materials. Among them, the most widely studied animal-derived natural antibacterial material is chitosan [44]. Jiao et al. [45] used physically crosslinked a polyamidoxime (PAO)–chitosan (CS) hybrid hydrogel membrane (PAO@CHM). The inhibition rates of PAO@CHM (Figure 3) against the tested strains were 70.86% (*Bacillus subtilis*), 72.34% (*Escherichia coli*), 94.20% (*Staphylococcus aureus*), and 96.03% (*Vibrio alginolyticus*). Compared with the filtered (sterile) seawater, the uptake of uranium by PAO@CHM (mCS:mPAO = 4:3) decreased in the nonfiltered (bacteria-containing) seawater at 8, 16, and 32 ppm for 5 days, respectively, at 12.31%, 8.87%, and 9.94%. The uranium-extraction capacity of PAO@CHM after 28 days of exposure to 500 L natural seawater reached 7.46 mg/g, which was higher than most reported PAO-based adsorbents and antibiofouling adsorbents.

The natural melanin in the cuttlefish ink sac has strong near-infrared absorption and efficient and fast photothermal conversion. Because natural melanin has the advantages of natural biocompatibility, biodegradability, and a high-photothermal-conversion efficiency, it is widely used in the medical field to help kill malignant tumors [46]. At the same time, squid ink extract has been shown to have good antibacterial activity in complex marine environments and is harmless to humans. Wang et al. [47] applied cuttlefish ink to polyamidoxime (PAO) hydrogel and designed a cuttlefish-ink-loaded polyamidoxime (CI-PAO) adsorbent with the dual functions of light and heat, as well as being antibacterial, for the efficient capture of uranium from natural seawater. The solar-energy-absorption rate of the CI-PAO film was 79.07%, which was 3.54 times that of the PAO film. Under single-sunlight conditions, the extraction capacity of the CI-PAO membrane in 500 mL of 8 ppm uranium-spiked simulated seawater was 488.76 mg/g, and the adsorption was basically completed after 16 h, which was 1.24 times that of the PAO membrane. After recycling five times, the uranium-adsorption capacity of CI-PAO still remained at 334.72 mg/g (72.81% of the initial adsorption capacity). CI-PAO exhibited an inhibition rate of about 75% in complex marine bacteria. After 4 weeks of immersion in natural seawater, the light-irradiated CI-PAO had a high-uranium-absorption capacity of 6.17 mg/g.

Plant-derived antibacterial materials use the bactericidal active substances of plants to achieve antibacterial purposes. As a natural, environmentally friendly, and degradable antibacterial agent, plant materials have a wide range of applications in food, biomedicine, and other fields [48]. Pu-erh tea extract has potential bacteriostatic activity against *Staphylococcus aureus* and *Bacillus subtilis*. Caffeine and epicatechins, the main components of tea polyphenols, have antimutagenic and antibacterial effects on these microorganisms [49]. Li et al. [50] prepared a green-tea waste/graphene aerogel (GDT) with photothermal conversion properties using the one-step hydrothermal method for uranium capture. The synergy between the GDT and tea residue had a good photothermal conversion performance, which increased the adsorption capacity of the GDT in natural seawater by 1.22 times. GDT has a capacity of 11.9 mg/g to extract uranium in natural seawater for 30 days and has a high selectivity. GDT had a good inhibitory effect on *Escherichia coli* and *Staphylococcus aureus*, with disinfection rates of 79.3% and 84.8%, respectively.

### 2.4. Antibiotic Antibacterial Materials

**Figure 4:**
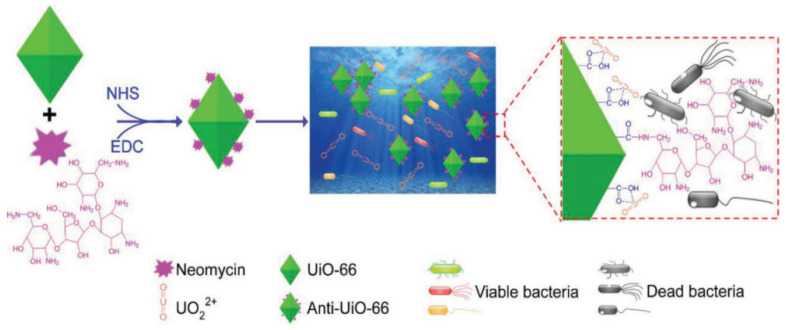
Schematic diagram of the structure and function of antibacterial adsorbent Anti-UiO-66 for seawater-uranium recovery [52].

Antibiotic antibacterial materials mainly refer to materials prepared from derivatives of microorganisms, which can be prepared by extraction and chemical synthesis. Neomycin is a widely used aminoglycoside antibiotic that inhibits bacteria by inhibiting protein synthesis or causing bacterial cell membrane collapse. The antibiotic neomycin shows broad-spectrum antibacterial activity against Gram-positive (G^+^) bacteria, Gram-negative (G^−^) bacteria, and mycobacteria. Because the biofouling bacteria that predominate in uranium-recovery sorbents are unknown, broad-spectrum antibiotics are the first choice for constructing antibiofouling sorbents for uranium recovery [51]. Yu et al. [52] covalently crosslinked neomycin with the carboxyl group on the MOF adsorbent UiO-66 to obtain Anti-UiO-66, an antibiological pollution uranium adsorbent material (Figure 4). Anti-UiO-66 has broad antimicrobial properties and can inhibit the growth of G^+^ and G^−^ bacteria. The growth inhibition rate of Anti-UiO-66 on marine bacteria was 87.03%. The uranium absorption of Anti-UiO-66 was 35.74% higher than that of UiO-66 in uranium-supplemented seawater containing 10^2^ CFU/mL bacteria. As the concentration of bacteria increased, which actually occurred in long-term field tests, the uranium-absorption capacity decreased further. In unfiltered 8 ppm uranium-spiked seawater, the uranium-absorption capacity of Anti-UiO-66 was as high as 284.45 ± 4.56 mg/g, which was 16.77% higher than that of UiO-66 at 244.25 ± 5.56 mg/g. After repeated use for five cycles, only an average decrease of 0.9575% in antibacterial activity was observed, indicating that tight covalent crosslinking is more stable for constructing antibiofouling sorbents. Immersed in natural seawater for 30 days, the uranium-absorption capacity of Anti-UiO-66 was 4.62 ± 0.09 mg/g, which was 24.4% higher than that of UiO-66.

The characteristics of seawater-uranium-extraction materials used in the ocean are different, and the main biological pollution bacteria are also different. Due to the extensive use of broad-spectrum antibiotics, a large number of drug-resistant strains and mutant strains have emerged, making the synthetic antibiofouling adsorption materials unable to inhibit the growth of all marine bacteria [53]. Therefore, the development of antibacterial compounds targeting specific biofouling bacteria may become a more effective means of antibiofouling.

### 2.5. Inorganic–Organic Hybrid Antibacterial Materials

With the deepening of the research on uranium-extraction materials in seawater, researchers have developed inorganic–organic hybrid materials with nanostructures, which have become promising candidates for uranium adsorbents due to their ease of preparation, high porosity, large specific surface area, abundant active sites, and biocompatibility [54]. Such materials obtain good antibacterial and antialgal activity through the photocatalytic functionalization of photosensitive functional groups or their nanostructures [55]. Hybrid materials in aqueous solutions are excited under light conditions. After photocatalysis, there will be a large amount of superoxide radicals (•O_2_^−^) and hydroxyl radicals (•OH^−^) in the aqueous solution, which have strong oxidation and can oxidize and destroy the organic matter on the surface of bacteria, resulting in the death of bacteria [56,57].

**Figure 5:**
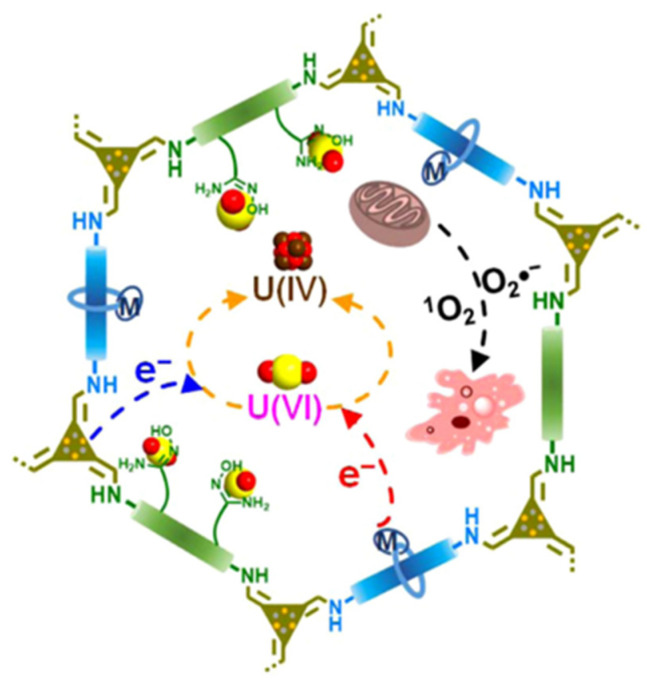
Schematic diagram of the multicomponent COF as an adsorption photocatalyst for uranium extraction from seawater [57].

Wei et al. [58] prepared highly planar conjugated naphthyl sp^2^-carbon-linked COF materials (NDA-TN-AO) with photocatalytic and photoelectric properties. NDA-TN-AO has high antibacterial activity against various strains tested, including Gram-positive *Bacillus subtilis*, Bacillus cereus, and *Staphylococcus aureus*, and Gram-negative *Escherichia coli*, *Vibrio alginolyticus*, and Pseudomonas aeruginosa. It can effectively kill bacteria and biological entities by producing biotoxic reactive oxygen species (ROS), and promote the photoelectron reduction of adsorbed U(VI) to insoluble U(IV), thereby significantly improving the extraction capacity of uranium. At a pH = 5.0, the uranium-adsorption capacity of NDA-TN-AO increased from 486.4 mg/g to 589.1 mg/g (an increase of 21.2%) after simulated sunlight irradiation. Due to the light-induced effect, the uranium-adsorption capacity of NDA-TN-AO in seawater reached 6.07 mg/g, which is 1.33 times that in the dark. Meng et al. [57] synthesized a multicomponent COF porous material (4-Pd-AO) containing amidoxime groups, triazine groups, and bipyridine metal groups. The •O_2_^−^ and ^1^O_2_ generated by the COF 4-Pd-AO under photocatalysis destroyed the cell walls of the bacteria and inhibited the growth of marine bacteria (Figure 5). The COF 4-Pd-AO had an inhibition rate of 96.86% against natural seawater under visible light irradiation, and 66.48% and 57.65% against algae Synechococcus elongatus and Chlorella under dark conditions, respectively. The COF 4-Pd-AO exhibited an extremely fast uranyl-capture capacity in spiked seawater samples, at about 25 ppb, removing 90% and 94% of uranyl within 30 min and 90 min, respectively. The value of the COF 4-Pd-AO extracted uranium from natural seawater under visible light irradiation was 4.62 mg/g per day.

Guo et al. [59] prepared a three-dimensional porous structure antibiofouling zeolite framework-8/chitosan/melamine sponge (CMZ8) adsorbent. The compressive stress of CMZ8 was 0.565 MPa and the algal cell death rate was 35%. At a pH = 8.0, the adsorption capacity of CMZ8 was 129.90 mg/g, and the adsorption capacity was 95.47 mg/g at the fifth adsorption–desorption cycle, which was higher than 91.45 mg/g of the ZIF-8 powder. For the adsorption of low-concentration U(VI), the absorption of U(VI) by the CMZ8 sponge reached 11.39 μg/g, and the removal rate of U(VI) was 65.45%. For the adsorption of the CMZ8 sponge in simulated polluted seawater, the removal rate of the CMZ8 at different initial U(VI) concentrations (3.473 to 206.3 μg/L) reached 70%.

## 3. Antiadhesion Materials

In addition to antimicrobial materials, inhibiting bioattachment by changing the properties of materials is also an important way to reduce biofouling. An effective way to prevent the adhesion of marine organisms is to prevent surface adhesion by adding hydrophilic or hydrophobic functional groups to make the adsorption material superhydrophilic or superhydrophobic because, when the water contact angle is 0° or 180°, the adhesion of marine organisms is close to 0. When the material exhibits superhydrophilic properties, water molecules are preferentially attached to the surface to form a thin liquid layer, which acts as a shield and terminates biological adhesion. When the surface energy of the material is reduced, it exhibits hydrophobic properties. By extending the distance between the surface and the fouling molecules, the material increases the kinetic barrier that the attached microorganisms need to overcome to reach the surface, so that the material exhibits an excellent antibiofouling ability [60,61].

Bai et al. [62] prepared a robust montmorillonite–polydopamine/polyacrylamide (MMT-PDA/PAM) nanocomposite hydrogel by a two-step method. The MMT_10_-PDA_0.6_/PAM-hydrogel-adsorption capacity can still reach 200.45 mg/g at a pH = 8.0. The maximum adsorption capacity of MMT_10_-PDA_0.6_/PAM reached 617.28 mg/g within 30 min (pH = 6.5, 400.0 ppm), which was higher than that of conventional hydrogel adsorbents. The MMT_10_-PDA_0.6_/PAM reached the equilibrium capacity after 30 min. In the flow-column system, after the adsorbent was in contact with the simulated seawater for 63 days, the uranium-absorption capacity from the simulated seawater reached 44 mg/g. After the sixth adsorption cycle, the uranium-absorption capacity of the recovered adsorbent was still 204.71 mg/g. The antiadhesion activity of MMT_10_-PDA_0.6_/PAM effectively prevented the attachment of Nitzschia after 8 days of exposure, and did not inhibit the growth of Nitzschia. The adsorption of uranium in the simulated seawater containing algae was basically not affected, and the adsorption amount could reach 2130 μg/g. Yuan and coworkers [63] introduced positively charged tetraethylenepentamine (TEPA) into negatively charged polyacrylamidoxime (PAO) to prepare a charged balanced hydrogel adsorbent. The as-prepared PAO-CB hydrogel membrane exhibited a strong antiadhesion ability against both marine biofouling and abiotic fouling, which was attributed to the reduced charge attraction between the adsorbent and the charged pollutants. Compared with the PAO membrane, the hydrophilicity of PAO-CB was increased because the crosslinked TEPA contained amino groups that could form hydrogen bonds with water. The characterization analysis of the PAO-CB membrane showed that, compared with the PAO membrane, the specific surface area, mechanical strength, and hydrophilicity of the adsorbent were all improved, which is beneficial to the uranium-extraction performance of the adsorbent. In natural seawater, the uranium-extraction capacity of the adsorbent PAO-CB reached 8.56 mg/g within 56 days, which was 44.59% higher than that of the PAO adsorbent. Due to the stable covalent linkage structure between PAO and TEPA, the adsorbent PAO-CB has high reusability and antiadhesion ability after repeated use. Huang and coworkers used the ATRP method to graft hydrophilic monomer 2-methacryloyloxyethyl phosphorylcholine on polyvinyl chloride–chlorinated polyvinyl chloride fiber (PVC-CPVC) to prepare the PVC-PC adsorption material [64]. The material had good hydrophilicity and antibioadhesion, but the monomer-grafting rate of the material was only 50%, resulting in a simulated saturated adsorption capacity of only 424.5 mg/g. Due to the complex preparation process of the nanomaterials, the technology is not mature enough, and the adsorption performance of the prepared materials needs to be improved, and significant research is still needed.

## 4. Challenges and Future Perspectives

The extraction of natural uranium from seawater is an inevitable means to maintain the stable development of nuclear energy. In recent years, researchers have performed significant research on the antibiofouling properties of seawater-uranium-extraction technology, but still face many challenges. The adsorption materials currently being studied target specific marine microorganisms and cannot overcome the serious hazards of various marine biological entities. The lack of tight bonds between inorganic antimicrobials and sorbents makes it impossible to use for long periods of time in complex marine environments. The combination of a single antibacterial substance and adsorption material is difficult to meet the requirements for antibacterial and adsorption, and a variety of materials need to be organically combined to achieve the expected goal [65,66,67]. At the same time, the experiment is not comprehensive, most of the research results at this stage are still under laboratory conditions, and there is less research on real seawater.

In view of the current research status of seawater-uranium extraction resistant to biological fouling, future research needs to pay attention to the following aspects. First of all, it is necessary to develop materials with excellent comprehensive properties with a wide range of antibacterial spectra to cope with the effects of biological fouling, pH, and ionic selectivity on the adsorption performance in the ocean, so as to achieve the purpose of improving the adsorption performance and selectivity in seawater environments. Second, the control the economic cost of materials research and development. The development of future materials will require a greater focus on simplifying procedures and establishing shorter and cheaper synthetic routes. While ensuring the stability and reusability of materials, combined with certain sea-trial experiments, the engineering application of seawater-uranium-extraction technology can be realized.

## 5. Conclusions

In order to cope with the shortage of uranium resources, seawater-uranium-extraction technology is an important strategy to cope with the sustainable development of human resources. Due to the complex marine environment, how to prepare antifouling adsorption materials with excellent comprehensive performance and improve the adsorption capacity is the focus of research in the field of seawater-uranium extraction. In recent years, there has been more and more research on seawater-uranium-extraction materials resistant to biofouling. Under the premise of satisfying certain antibacterial and antiadhesion needs, the adsorption capacity of the material has been significantly improved, but there is still a large gap in the engineering application of seawater-uranium extraction. Therefore, when researchers study new materials, they need to pay more attention to the selection of green, new, nontoxic, and biocompatible substances. Improving the service life of materials in real-sea areas, and designing and preparing biofouling-resistant seawater-uranium-extraction materials with stronger marine applicability, will be the main problems that need to be solved in the future. It is believed that, through the joint efforts of scientific researchers to find solutions to these problems, this will bring about great developments in the field of seawater-uranium extraction.

## Data Availability

Not applicable.

## References

[1] Yue X.H., Peng M.Y.P., Anser M.K., Nassani A.A., Haffar M., Zaman K. (2022). The role of carbon taxes, clean fuels, and renewable energy in promoting sustainable development: How green is nuclear energy?. Renew. Energy.

[2] Luo W., Xiao G., Tian F., Richardson J.J., Wang Y.P., Zhou J.F., Guo J.L., Liao X.P., Shi B. (2019). Engineering robust metal-phenolic network membranes for uranium extraction from seawater. Energy Environ. Sci..

[3] Kim J., Tsouris C., Mayes R.T., Oyola Y., Saito T., Janke C.J., Dai S., Schneider E., Sachde D. (2013). Recovery of Uranium from Seawater: A Review of Current Status and Future Research Needs. Sep. Sci. Technol..

[4] Guo H., Mei P., Xiao J.T., Huang X.S., Ishag A., Sun Y.B. (2021). Carbon materials for extraction of uranium from seawater. Chemosphere.

[5] Zhao S.L., Yuan Y.H., Yu Q.H., Niu B.Y., Liao J.H., Guo Z.H., Wang N. (2019). A Dual-Surface Amidoximated Halloysite Nanotube for High-Efficiency Economical Uranium Extraction from Seawater. Angew. Chem. -Int. Ed..

[6] Li P., Chen P., Wang G.H., Wang L.Z., Wang X.G., Li Y.R., Zhang W.M., Jiang H., Chen H. (2020). Uranium elimination and recovery from wastewater with ligand chelation-enhanced electrocoagulation. Chem. Eng. J..

[7] Shi S., Qian Y.X., Mei P.P., Yuan Y.H., Jia N., Dong M.Y., Fan J.C., Guo Z.H., Wang N. (2020). Robust flexible poly(amidoxime) porous network membranes for highly efficient uranium extraction from seawater. Nano Energy.

[8] Amphlett J.T.M., Ogden M.D., Foster R.I., Syna N., Soldenhoff K.H., Sharrad C.A. (2018). The effect of contaminants on the application of polyamine functionalised ion exchange resins for uranium extraction from sulfate based mining process waters. Chem. Eng. J..

[9] Li P., Wang J.J., Wang Y., Dong L., Wang W., Geng R.Y., Ding Z., Luo D.X., Pan D.Q., Liang J.J. (2021). Ultrafast recovery of aqueous uranium: Photocatalytic U(VI) reduction over CdS/g-C3N4. Chem. Eng. J..

[10] Tan L.C., Liu Q., Jing X.Y., Liu J.Y., Song D.L., Hu S.X., Liu L.H., Wang J. (2015). Removal of uranium(VI) ions from aqueous solution by magnetic cobalt ferrite/multiwalled carbon nanotubes composites. Chem. Eng. J..

[11] Ling C.J., Liu X.Y., Yang X.J., Hu J.T., Li R., Pang L.J., Ma H.J., Li J.Y., Wu G.Z., Lu S.M. (2017). Uranium Adsorption Tests of Amidoxime-Based Ultrahigh Molecular Weight Polyethylene Fibers in Simulated Seawater and Natural Coastal Marine Seawater from Different Locations. Ind. Eng. Chem. Res..

[12] Wu Y.D., Cui W.R., Zhang C.R., Liang R.P., Qiu J.D. (2021). Regenerable, anti-biofouling covalent organic frameworks for monitoring and extraction of uranium from seawater. Environ. Chem. Lett..

[13] Gill G.A., Kuo L.J., Janke C.J., Park J., Jeters R.T., Bonheyo G.T., Pan H.B., Wai C., Khangaonkar T., Bianucci L. (2016). The Uranium from Seawater Program at the Pacific Northwest National Laboratory: Overview of Marine Testing, Adsorbent Characterization, Adsorbent Durability, Adsorbent Toxicity, and Deployment Studies. Ind. Eng. Chem. Res..

[14] Yuan Y.H., Yu Q.H., Cao M., Feng L.J., Feng S.W., Liu T.T., Feng T.T., Yan B.J., Guo Z.H., Wang N. (2021). Selective extraction of uranium from seawater with biofouling-resistant polymeric peptide. Nat. Sustain..

[15] Xu X., Xu L., Ao J.X., Liang Y.L., Li C., Wang Y.J., Huang C., Ye F., Li Q.N., Guo X.J. (2020). Ultrahigh and economical uranium extraction from seawater via interconnected open-pore architecture poly(amidoxime) fiber. J. Mater. Chem. A.

[16] Park J., Gill G.A., Strivens J.E., Kuo L.J., Jeters R.T., Avila A., Wood J.R., Schlafer N.J., Janke C.J., Miller E.A. (2016). Effect of Biofouling on the Performance of Amidoxime-Based Polymeric Uranium Adsorbents. Ind. Eng. Chem. Res..

[17] Lejars M., Margaillan A., Bressy C. (2012). Fouling Release Coatings: A Nontoxic Alternative to Biocidal Antifouling Coatings. Chem. Rev..

[18] Yebra D.M., Kiil S., Dam-Johansen K. (2004). Antifouling technology—Past, present and future steps towards efficient and environmentally friendly antifouling coatings. Prog. Org. Coat..

[19] Delauney L., Compere C., Lehaitre M. (2010). Biofouling protection for marine environmental sensors. Ocean Sci..

[20] Qiu H.Y., Feng K., Gapeeva A., Meurisch K., Kaps S., Li X., Yu L.M., Mishra Y.K., Adelung R., Baum M. (2022). Functional polymer materials for modern marine biofouling control. Prog. Polym. Sci..

[21] Esmeryan K.D., Chaushev T.A. (2023). Anti-biofouling potential of extremely water-repellent carbon soot coatings immersed in a highly-contaminated seawater swamp. Prog. Org. Coat..

[22] Zhang X.C., Zhang Z.C., Shu Q.M., Xu C., Zheng Q.Q., Guo Z., Wang C., Hao Z.X., Liu X., Wang G.Q. (2021). Copper Clusters: An Effective Antibacterial for Eradicating Multidrug-Resistant Bacterial Infection In Vitro and In Vivo. Adv. Funct. Mater..

[23] Xie Q.Y., Pan J.S., Ma C.F., Zhang G.Z. (2019). Dynamic surface antifouling: Mechanism and systems. Soft Matter.

[24] Gu Y.Q., Yu L.Z., Mou J.G., Wu D.H., Xu M.S., Zhou P.J., Ren Y. (2020). Research Strategies to Develop Environmentally Friendly Marine Antifouling Coatings. Mar. Drugs.

[25] Jin H.C., Tian L.M., Bing W., Zhao J., Ren L.Q. (2022). Bioinspired marine antifouling coatings: Status, prospects, and future. Prog. Mater. Sci..

[26] Ma D.S., Xu X., Li Z.W., Peng H., Cai D., Wang D., Yue Q. (2022). Nanoemulsion assembly toward vaterite mesoporous CaCO3 for high-efficient uranium extraction from seawater. J. Hazard. Mater..

[27] Bouazizi N., Vieillard J., Samir B., Le Derf F. (2022). Advances in Amine-Surface Functionalization of Inorganic Adsorbents for Water Treatment and Antimicrobial Activities: A Review. Polymers.

[28] Hajipour M.J., Fromm K.M., Ashkarran A.A., de Aberasturi D.J., de Larramendi I.R., Rojo T., Serpooshan V., Parak W.J., Mahmoudi M. (2012). Antibacterial properties of nanoparticles. Trends Biotechnol..

[29] Meikle T.G., Dyett B.P., Strachan J.B., White J., Drummond C.J., Conn C.E. (2020). Preparation, Characterization, and Antimicrobial Activity of Cubosome Encapsulated Metal Nanocrystals. Acs Appl. Mater. Interfaces.

[30] Mokabber T., Cao H.T., Norouzi N., van Rijn P., Pei Y.T. (2020). Antimicrobial Electrodeposited Silver-Containing Calcium Phosphate Coatings. Acs Appl. Mater. Interfaces.

[31] Pu Y.D., Qiang T.T., Ren L.F. (2022). Anti-biofouling bio-adsorbent with ultrahigh uranium extraction capacity: One uranium resource recycling solution. Desalination.

[32] Sun W.Y., Feng L.J., Zhang J.C., Lin K., Wang H., Yan B.J., Feng T.T., Cao M., Liu T., Yuan Y.H. (2022). Amidoxime Group-Anchored Single Cobalt Atoms for Anti-Biofouling during Uranium Extraction from Seawater. Adv. Sci..

[33] Chen D.Y., Zhao X.Y., Jing X.F., Zhao R., Zhu G.S., Wang C. (2023). Bio-inspired functionalization of electrospun nanofibers with anti-biofouling property for efficient uranium extraction from seawater. Chem. Eng. J..

[34] Sha D., Xu J.D., Yang X., Xue Y.H., Liu X., Li C.X., Wei M., Liang Z.W., Shi K., Wang B.L. (2021). Synthesis and antibacterial activities of quaternary ammonium salts with different alkyl chain lengths grafted on polyvinyl alcohol-formaldehyde sponges. React. Funct. Polym..

[35] Valls A., Andreu J.J., Falomir E., Luis S.V., Atrian-Blasco E., Mitchell S.G., Altava B. (2020). Imidazole and Imidazolium Antibacterial Drugs Derived from Amino Acids. Pharmaceuticals.

[36] Han H., Zhu J., Wu D.Q., Li F.X., Wang X.L., Yu J.Y., Qin X.H. (2019). Inherent Guanidine Nanogels with Durable Antibacterial and Bacterially Antiadhesive Properties. Adv. Funct. Mater..

[37] Li P., Sun S.Y., Dong A., Hao Y.P., Shi S.Q., Sun Z.J., Gao G., Chen Y.X. (2015). Developing of a novel antibacterial agent by functionalization of graphene oxide with guanidine polymer with enhanced antibacterial activity. Appl. Surf. Sci..

[38] He N.N., Li H., Li L.Y., Cheng C., Lu X.R., Wen J., Wang X.L. (2021). Polyguanidine-modified adsorbent to enhance marine applicability for uranium recovery from seawater. J. Hazard. Mater..

[39] Zhao Y., Li J.W., Wu S.J., Cheng H.M. (2022). Ion-imprinted guanidine-functionalized zeolite molecular sieves enhance the adsorption selectivity and antibacterial properties for uranium extraction. Rsc Adv..

[40] Zhang W., Bu A., Ji Q., Min L., Zhao S., Wang Y., Chen J. (2019). pK(a) -Directed Incorporation of Phosphonates into MOF-808 via Ligand Exchange: Stability and Adsorption Properties for Uranium. Acs Appl. Mater. Interfaces.

[41] Zhao Y.L., Dai L., Zhang Q.F., Zhang S.B. (2019). Surface modification of polyamide reverses osmosis membrane by phosphonic acid group with improved performance. J. Appl. Polym. Sci..

[42] He Y.C., Hou G.S., Lu X.R., Chang P.P., Shao D.D. (2022). Application of poly(vinylphosphonic acid) modified poly(amidoxime) in uptake of uranium from seawater. Rsc Adv..

[43] Silver L.L. Natural product screening for antibacterial agents. Proceedings of the 1st International Symposium on Natural Preservatives in Food Systems.

[44] Li J.H., Zhuang S.L. (2020). Antibacterial activity of chitosan and its derivatives and their interaction mechanism with bacteria: Current state and perspectives. Eur. Polym. J..

[45] Jiao G.J., Ma J.L., Zhang J.Q., Li Y.C., Liu K.N., Sun R.C. (2022). Porous and biofouling-resistant amidoxime-based hybrid hydrogel with excellent interfacial compatibility for high-performance recovery of uranium from seawater. Sep. Purif. Technol..

[46] Zhang J.L., Shi C.Z., Shan F., Shi N.N., Ye W., Zhuo Y.Y., Zhang Y.J., Zhang Z.Y., Shi Y.X., Peng C. (2021). From biology to biology: Hematoporphyrin-melanin nanoconjugates with synergistic sonodynamic-photothermal effects on malignant tumors. Chem. Eng. J..

[47] Wang H., Xu T.H., Zheng B.H., Cao M., Gao F., Zhou G.B., Ma C., Dang J., Yao W.K., Wu K.C. (2022). Cuttlefish ink loaded polyamidoxime adsorbent with excellent photothermal conversion and antibacterial activity for highly efficient uranium capture from natural seawater. J. Hazard. Mater..

[48] Su Y., Zhang C., Wang Y., Li P. (2012). Antibacterial property and mechanism of a novel Pu-erh tea nanofibrous membrane. Appl. Microbiol. Biotechnol..

[49] Wu S.C., Yen G.C., Wang B.S., Chiu C.K., Yen W.J., Chang L.W., Duh P.D. (2007). Antimutagenic and antimicrobial activities of pu-erh tea. Lwt-Food Sci. Technol..

[50] Li N., Wu J., Su R., Zhang N., Zhao J., Wang Z. (2023). Bioinspired green tea waste/graphene aerogel for solar-enhanced uranium extraction from seawater. Desalination.

[51] Rebitski E.P., Alcantara A.C.S., Darder M., Cansian R.L., Gomez-Hortiguela L., Pergher S.B.C. (2018). Functional Carboxymethylcellulose/Zein Bionanocomposite Films Based on Neomycin Supported on Sepiolite or Montmorillonite Clays. Acs Omega.

[52] Yu Q.H., Yuan Y.H., Wen J., Zhao X.M., Zhao S.L., Wang D., Li C.Y., Wang X.L., Wang N. (2019). A Universally Applicable Strategy for Construction of Anti-Biofouling Adsorbents for Enhanced Uranium Recovery from Seawater. Adv. Sci..

[53] Yan L., Gopal A., Kashif S., Hazelton P., Lan M.H., Zhang W.J., Chen X.F. (2022). Metal organic frameworks for antibacterial applications. Chem. Eng. J..

[54] Wang L.L., Luo F., Dang L.L., Li J.Q., Wu X.L., Liu S.J., Luo M.B. (2015). Ultrafast high-performance extraction of uranium from seawater without pretreatment using an acylamide- and carboxyl-functionalized metal-organic framework. J. Mater. Chem. A.

[55] Liu C., Wang W., Zhang M.T., Zhang C.Y., Ma C.C., Cao L., Kong D.B., Feng H.M., Li W., Chen S.G. (2022). Synthesis of MXene/COF/Cu_2_O heterojunction for photocatalytic bactericidal activity and mechanism evaluation. Chem. Eng. J..

[56] Zhong X., Liu Y.X., Zeng W.X., Zhu Y.L., Hu B.W. (2022). Excellent photoreduction performance of U(VI) on metal organic framework/covalent organic framework heterojunction by solar-driven. Sep. Purif. Technol..

[57] Hao M.J., Chen Z.S., Liu X.L., Liu X.H., Zhang J.Y., Yang H., Waterhouse G.I.N., Wang X.K., Ma S.Q. (2022). Converging Cooperative Functions into the Nanospace of Covalent Organic Frameworks for Efficient Uranium Extraction from Seawater. Ccs Chem..

[58] Cui W.R., Li F.F., Xu R.H., Zhang C.R., Chen X.R., Yan R.H., Liang R.P., Qiu J.D. (2020). Regenerable Covalent Organic Frameworks for Photo-enhanced Uranium Adsorption from Seawater. Angew. Chem. -Int. Ed..

[59] Guo X.J., Yang H.C., Wang J. (2022). Ion cross-linking assisted synthesis of ZIF-8/chitosan/melamine sponge with anti-biofouling activity for enhanced uranium recovery. Inorg. Chem. Front..

[60] Esmeryan K.D., Castano C.E., Abolghasemibizaki M., Mohammadi R. (2017). An artful method for in-situ assessment of the anti-biofouling potential of various functional coatings using a quartz crystal microbalance. Sens. Actuators B-Chem..

[61] Cui Y., Yuan W.Q. (2013). Thermodynamic modeling of algal cell-solid substrate interactions. Appl. Energy.

[62] Bai Z.Y., Liu Q., Zhang H.S., Liu J.Y., Chen R.R., Yu J., Li R.M., Liu P.L., Wang J. (2020). Mussel-inspired anti-biofouling and robust hybrid nanocomposite hydrogel for uranium extraction from seawater. J. Hazard. Mater..

[63] Yuan Y.H., Guo X., Feng L.J., Yu Q.H., Lin K., Feng T.T., Yan B.J., Fedorovich K.V., Wang N. (2021). Charge balanced anti-adhesive polyacrylamidoxime hydrogel membrane for enhancing uranium extraction from seawater. Chem. Eng. J..

[64] Huang Z., Dong H., Yang N., Li H., He N.N., Lu X.R., Wen J., Wang X.L. (2020). Bifunctional Phosphorylcholine-Modified Adsorbent with Enhanced Selectivity and Antibacterial Property for Recovering Uranium from Seawater. Acs Appl. Mater. Interfaces.

[65] Li H., He N.N., Cheng C., Dong H., Wen J., Wang X.L. (2020). Antimicrobial polymer contained adsorbent: A promising candidate with remarkable anti-biofouling ability and durability for enhanced uranium extraction from seawater. Chem. Eng. J..

[66] Wu Y., Xie Y.H., Liu X.L., Li Y., Wang J.Y., Chen Z.S., Yang H., Hu B.W., Shen C., Tang Z.W. (2023). Functional nanomaterials for selective uranium recovery from seawater: Material design, extraction properties and mechanisms. Coord. Chem. Rev..

[67] Ielo I., Giacobello F., Castellano A., Sfameni S., Rando G., Plutino M.R. (2022). Development of Antibacterial and Antifouling Innovative and Eco-Sustainable Sol-Gel Based Materials: From Marine Areas Protection to Healthcare Applications. Gels.

